# Physiotherapy practice in the private sector: organizational characteristics and models

**DOI:** 10.1186/1472-6963-14-362

**Published:** 2014-08-29

**Authors:** Kadija Perreault, Clermont E Dionne, Michel Rossignol, Stéphane Poitras, Diane Morin

**Affiliations:** Centre for Interdisciplinary Research in Rehabilitation and Social Integration (CIRRIS), Institut de réadaptation en déficience physique de Québec, Québec City, Canada; Université Laval, Québec City, Canada; Axe Santé des populations et Pratiques optimales en santé, CHU de Québec Research Center, Hôpital du St-Sacrement, 1050 Chemin Sainte-Foy, Québec City, G1S 4L8 Canada; Institut national d’excellence en santé et en services sociaux, Montréal, Canada; Department of Epidemiology, Biostatistics and Occupational Health, McGill University, Montréal, Canada; School of Rehabilitation Sciences, Faculty of Health Sciences, University of Ottawa, Ottawa, Canada; Institut universitaire de formation et de recherche en soins, Université de Lausanne, Lausanne, Switzerland

**Keywords:** Organizational characteristics, Organizational models, Physiotherapy, Private sector, Survey

## Abstract

**Background:**

Even if a large proportion of physiotherapists work in the private sector worldwide, very little is known of the organizations within which they practice. Such knowledge is important to help understand contexts of practice and how they influence the quality of services and patient outcomes. The purpose of this study was to: 1) describe characteristics of organizations where physiotherapists practice in the private sector, and 2) explore the existence of a taxonomy of organizational models.

**Methods:**

This was a cross-sectional quantitative survey of 236 randomly-selected physiotherapists. Participants completed a purpose-designed questionnaire online or by telephone, covering organizational vision, resources, structures and practices. Organizational characteristics were analyzed descriptively, while organizational models were identified by multiple correspondence analyses.

**Results:**

Most organizations were for-profit (93.2%), located in urban areas (91.5%), and within buildings containing multiple businesses/organizations (76.7%). The majority included multiple providers (89.8%) from diverse professions, mainly physiotherapy assistants (68.7%), massage therapists (67.3%) and osteopaths (50.2%). Four organizational models were identified: 1) solo practice, 2) middle-scale multiprovider, 3) large-scale multiprovider and 4) mixed.

**Conclusions:**

The results of this study provide a detailed description of the organizations where physiotherapists practice, and highlight the importance of human resources in differentiating organizational models. Further research examining the influences of these organizational characteristics and models on outcomes such as physiotherapists’ professional practices and patient outcomes are needed.

## Background

In Canada, like in many parts of the world, physiotherapists work in the private and public sectors. According to the Canadian Institute for Health Information [1], the *public sector* includes “employees working within government and government institutions, such as hospitals, schools and universities”. Canada has a national health insurance program that is designed to ensure reasonable access to medically necessary hospital and physician services [2]. The thirteen Canadian provinces and territories are responsible for the management, organization and delivery of health services for their residents under the Canada Health Act [2]. Physiotherapy services offered in hospitals are usually publicly funded, while there is variability between provinces and territories for community services [3]. As for the private sector, it includes “employees working within privately owned facilities, organizations and businesses, and third-party insurers, self-employed private practitioners and owners of a business.” Physiotherapy services received in this sector are funded by the service users themselves or via third party payers (e.g. insurance companies or workers compensation boards). In 2011, the proportion of Canadian physiotherapists working in the private sector was 42.3% [1]. Private sector physiotherapy is also reality on all continents [4].

The private sector is often central in offering physiotherapy services for persons presenting musculoskeletal conditions. For example, in Québec, the second most populated province of Canada, the great majority of workers presenting with low back pain receives services in the private sector [5]. Because many physiotherapists also practice legally in direct access worldwide (without medical reference) [4], private sector physiotherapists represent first line primary care professionals to whom populations seek treatment from, especially in contexts where access to publicly funded physiotherapy services is limited, such as in Canada [3, 6, 7].

In the last few years, although an increasing number of studies have examined the clinical practices of physiotherapists, only a small number have focussed on practice in the private sector (e.g. [5, 8–11]). Furthermore, very little is known of the organizations within which private sector physiotherapists practice (their workplaces), merely a few studies having reported on this subject (e.g. [12, 13]). Gaining further knowledge on these organizations would help understand the contexts in which these physiotherapists work and help assess if and how these organizational-level elements influence the quality of interventions and patient outcomes. The results would also provide useful information for various stakeholders involved in the development of the physiotherapy workforce, including professional boards and associations.

Using a configurational approach has been suggested in recent years to analyze such healthcare organizations. According to this approach, organizations are characterized by interrelated features that form context-dependent configurations [14, 15], viewed as holistic entities [16, 17]. Identifying such configurations helps to understand relationships between organizational characteristics [18], as well as to provide a means of assessing organizational performance. This contrasts with traditional methods of evaluating impacts of organizational models focusing only on individual features of organizations [16].

Previous studies using configurational approaches have focused on different dimensions or organizational components, such as organizational differentiation, integration and centralization [19], staffing, scope of practice and work environment [20], or processes, structure, environmental factors and strategy [18]. Lamarche et al. [14] analyzed primary care medical practices based on four organizational components: 1) the organization’s *vision*, that includes beliefs, representations, values and goals that allow actors to communicate and assign meaning to their actions, 2) its *resources*, that relate to the quantity and quality of resources used in carrying out activities, 3) its *structure*, which includes laws, rules, conventions, etc. that shape actors’ behaviors and relations between them, and 4) its *practices*, that represent the processes that lead to the production of activities, products and services. Two main types of configurations resulting from the configurational approach have been put forward: 1) typologies, which are conceptually defined classifications, and 2) taxonomies, that are classifications into relatively homogeneous groups of entities (such as organizations) derived from empirical work, using multivariate analyses [15, 21].

In light of the importance of private sector practice in the physiotherapy field and the lack of knowledge on the organizational contexts within which physiotherapists work in this sector, the objectives of the present study were to 1) describe the characteristics of organizations where physiotherapists practice in the private sector, and 2) explore the existence of a taxonomy of organizational models.

## Methods

### Study design

Data for this study were obtained through conducting a cross-sectional survey [22], that combined data collection through the telephone and internet. This survey was the second part of a larger two-part mixed-methods study that aimed at drawing the portrait of the interprofessional practices of physiotherapists working in the private sector with adults with low back pain. The first part of this larger study was a qualitative descriptive study that contributed to the development of the questionnaire used in the present survey. The Ethics Committee of the *Institut de réadaptation en déficience physique de Québec* approved this study (# 2010–190).

### Selection of participants

Practicing physiotherapists acted as respondents for the current study. Potential respondents were selected using simple random sampling from the list of 966 physiotherapists who had accepted to be contacted for research purposes (in the annual membership renewal form) out of the 1566 physiotherapists who, as of April 2011, were members of the Order of Physiotherapy of the Province of Québec (OPPQ - Order membership being mandatory to practice as a physiotherapist in Québec) and worked in the private sector. To be included in the study, the physiotherapists also had to: 1) practice at least one day/week, 2) have a clientele comprising at least 20% of people consulting for low back pain, 3) have been working in the same organization in the previous three months, 4) mainly provide interventions to adults, 5) not be off work at the time of the study, and 6) accept to complete a questionnaire in French, Québec having a population of over eight million inhabitants, a majority of them speaking French. Physiotherapists off work at the time of the study (e.g. maternity leave, illness) were excluded. The sample size required for our overall survey was estimated at 309 randomly selected physiotherapists. Because the main objectives of the current study related to the organizations the physiotherapists worked in, not the physiotherapists themselves, and that it was possible that more than one physiotherapist be randomly selected per organization to respond to the questionnaire, only data from the first randomly-selected physiotherapist per organization to have answered organizational-level questions in the questionnaire were retained for the analyses.

### Recruitment procedure and data collection

Potential participants were first contacted by telephone to explain the study and verify eligibility. Eligible and interested physiotherapists were then sent an email that gave them access to further information on the study and to the questionnaire, if they consented to participate. The survey questionnaire was constructed using the SurveyMonkey platform [23] and comprised 69 questions (plus sub-questions) covering the following areas: physiotherapists’ socio-demographic and professional profile (11 questions), their interprofessional practices (37 questions), as well as the characteristics of the organization they worked in (21 questions). These latter questions covered the four organizational components of Lamarche et al.’s [14] previously- mentioned configurational approach and provided the main data for the present study. Questionnaire construction was guided by development and testing recommendations [24, 25], our literature search including existing questionnaires [8, 26–33], and results of our previous qualitative study. Seven physiotherapists who did not participate in the study pre-tested the questionnaire. Data collection extended from July 2011 to January 2012.

### Data analyses

Survey data were first downloaded from the SurveyMonkey platform into Excel and SPSS spreadsheets and were then reviewed for exactness and missing/extreme values. To describe characteristics of organizations, descriptive statistics were carried out. In order to explore the existence of different organizational models, multiple correspondence analyses using frequency data of organizational variables were conducted [34–36]. This type of analysis allows to examine associations between nominal variables in a contingency table, as well as between categories of each variable [34–36]. It is also viewed as a graphic approach, as points relating to rows and columns of the contingency table are projected onto a bi-dimensional graph [35, 37]. It was used previously to examine practice and continuing education profiles of physiotherapists [5, 38].

**Figure 1:**
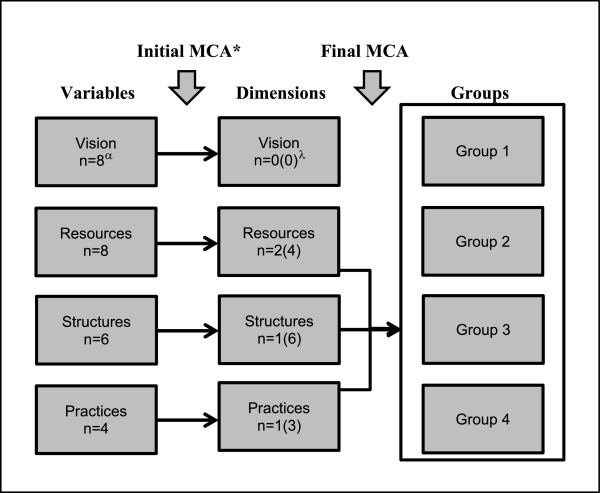
**Major steps in conducting multiple correspondence analyses.** *MCA = multiple correspondence analyses. ^α^In boxes in this column, numbers correspond to the number of variables included in initial MCA for each organizational component. ^λ^In boxes in this column, the first number indicates number of dimensions retained after initial MCA, while the second number (in parentheses) indicates the corresponding number of variables remaining following these initial analyses.

The first steps taken before conducting multiple correspondence analyses were to transform continuous variables into categorical variables, as well as to go over contingency tables to combine categories with small cell frequencies [35]. Cut-offs between categories were also based on the presence of a natural cut-off or the possibility to obtain relatively equal distributions between categories, as done in previous work [39]. Next, because of the relatively high number of variables and the difficulties this entails for analyses and interpretation of the visual representations [40], multiple correspondence analyses were conducted separately for variables associated with each organizational component of the configurational approach, a method used successfully previously [17, 39]. This led to the identification of significant dimensions to retain for each organizational component, based on percentage of explained variance, interpretability, as well as visual representation of variables and organizations [17, 35, 36]. Examining the bi-dimensional graphs allowed to appreciate proximity between points represented on either of the two dimensions, an indication of relationships between categories of variables [35], as well as the shape of the plots [41]. Non-contributory variables, as manifested by very close localizations of the categories of a variable on the graphs, were retained as supplementary variables in an attempt to help further interpretation [34]. Final multiple correspondence analyses were then conducted using the coordinates of each organization for the significant dimensions obtained by multiple correspondence analyses of each organizational component. Figure 1 gives an overview of these analyses for which list-wise deletion of missing data was carried out. SPSS [42] software was used to conduct all analyses.

## Results

### Study sample

Three hundred twenty-seven physiotherapists working in 243 different organizations in Québec responded to the survey questionnaire, for a 67.7% proportion of participation. Out of this sample, data from 11 physiotherapists were excluded because the respondents had answered none of the questions relating to organizational data. Of the 316 remaining questionnaires, by retaining only the data provided by the first physiotherapist respondent to have been randomly selected in each organization, we obtained data from 236 physiotherapists/organizations (72.2% of the total sample of 327 respondents).

### Characteristics of respondents and organizations

Table 1 presents socio-demographic and professional characteristics of the physiotherapists who responded to the questionnaire. Participants were mostly women (63.6%), had a Bachelor’s degree as their physiotherapy diploma (92.4%), and had 13.3 ± 9.8 (mean ± standard deviation) years of professional experience. A high proportion of their clientele presented with low back pain (average of 38.5% ± 14.0%) and they mostly reported using mechanical (55.5%) and functional/exercise approaches (50.0%), respectively as primary and secondary intervention approaches with this clientele.

**Table 1:** **Selected socio-demographic and professional characteristics of physiotherapists (n = 236, unless noted)**

Variable	n (%)	mean (SD)	missing n (%)
Gender			
Men	86 (36.4)		
Women	150 (63.6)		
Age (years; range 23.9-69.1)		38.4 (10.2)	
Mother tongue			
French	201 (85.2)		
English	20 (8.5)		
Other	15 (6.4)		
Main language used with patients			
French	216 (91.5)		
English	16 (6.8)		
Other	4 (1.7)		
Highest level of education			
Baccalaureate	189 (80.1)		
Certificate/micro-program	6 (2.5)		
Master’s	23 (9.7)		
Doctoral	1 (0.4)		
Other	17 (7.2)		
Level of education of PT^β^ diploma			1 (0.4)
Baccalaureate	218 (92.4)		
Master’s	14 (5.9)		
Other	3 (1.3)		
Professional experience (years)		13.3 (9.8)	
Professional experience with people with LBP^λ^ (years)		12.5 (9.4)	
Duration of work in organization (years)		8.1 (7.1)	
Affiliation with organization			
Owner/co-owner	81 (34.3)		
Self-employed	28 (11.9)		
Employee	127 (53.8)		
Remuneration			
Per patient (visit)	108 (45.8)		
Hourly salary	84 (35.6)		
Mixed (per patient + hourly)	24 (10.2)		
Other	20 (8.5)		
Worked also in another organization	54 (22.9)		2 (0.8)
Mean hours worked in a usual week*		33.1 (8.7)	2 (0.8)
Mean patients seen (visits) in a usual week*		48.7 (18.2)	9 (3.8)
Mean nights worked after 6 pm in a usual week*		1.6 (1.2)	11 (4.7)
Contributed to teaching in physiotherapy*	18 (7.6)		4 (1.7)
Supervised physiotherapy training*	58 (24.6)		
% of clientele with LBP*		38.5 (14.0)	2 (0.8)
% of clientele with LBP referred by physician*		40.7 (24.5)	1 (0.4)
% of clientele with LBP covered by*:			2 (0.8)
Workers’ Compensation Board		20.9 (18.3)	
Automobile Insurance Society		9.1 (9.6)	
Private insurance		56.3 (23.8)	
Person		12.4 (11.1)	
Other		1.3 (2.9)	
% of clientele according to stage of LBP*:			1 (0.4)
Acute		33.5 (19.5)	
Sub-acute		33.2 (14.1)	
Chronic		33.3 (19.3)	
Main intervention approach for patients with LBP:			1 (0.4)
Conventional^α^	25 (10.6)		
Functional/exercise	33 (14.0)		
Mechanical (McKenzie/manual therapy)	131 (55.5)		
Osteopathic/global postural re-education	41 (17.4)		
Other	5 (2.1)		
Secondary intervention approach for patients with LBP:			6 (2.5)
Conventional	56 (23.7)		
Functional/exercise	118 (50.0)		
Mechanical (McKenzie/manual therapy)	41 (17.4)		
Osteopathic/global postural re-education	14 (5.9)		
Other	1 (0.4)		

^β^PT = physiotherapy.

^λ^LBP = low back pain.

*In previous 12 months.

^α^Includes for example electrotherapy, physical modalities, soft tissue techniques.

Results of the characteristics of the organizations are found in Table 2. In terms of characteristics related to vision, most organizations were said to be for-profit (93.2%) and the perceived importance of organizational values, such as patients’ physical and psychosocial health, was rated highly (range between 7.3 ± 2.1 and 9.7 ± 0.7 on a 0–10 scale). In terms of resources, most organizations were located in an urban environment (91.5%) and more specifically, in buildings comprising multiple businesses/organizations (76.7%). The great majority of organizations where physiotherapists practiced included multiple providers (89.8%), such as physiotherapy assistants (68.7%), massage therapists (67.3%) and osteopaths (50.2%), but more rarely family physicians (8.1%) and chiropractors (2.4%), for a mean of 9.1 (SD = 6.5) providers per organization and 4.4 (SD = 2.6) different professions represented in each organization. Moreover, most organizations (64.0%) were in close physical proximity with other organizations where other providers worked. They also had secretaries working in the organizations (87.7%) and offered computer and internet access to physiotherapists (respectively 92.8% and 88.6%). A minority of organizations had vacant provider positions (27.5%) and electronic physiotherapy patient records (11.0%).

**Table 2:** **Selected characteristics of organizations (n = 236, unless noted)**

Variables	n (%)	mean (SD)	missing n (%)
*Vision*			
For-profit*	220 (93.2)		1 (0.4)
Importance* ^*α*^ *of:*			
Patients’ physical health		9.7 (0.7)	2 (0.8)
Patients’ psychological and social health		8.3 (1.9)	2 (0.8)
Prevention and health promotion		8.7 (1.5)	4 (1.7)
Research results		7.4 (2.1)	2 (0.8)
Financial profit		7.3 (2.1)	3 (1.3)
Respect, courtesy, confidentiality		9.5 (1.0)	4 (1.7)
Interactions between PTs^λ^ and other providers^χ^		8.2 (2.0)	2 (0.8)
*Resources*			
General location*			
Urban^ψ^	216 (91.5)		
Rural	20 (8.5)		
Specific location^β^			
PT’s home	10 (4.2)		
Building with organization only	26 (11.0)		
Building with multiple businesses/organizations	181 (76.7)		
Academic institution	7 (3.0)		
Other	12 (5.1)		
Number of providers^*β*^		9.1 (6.5)	4 (1.7)
1	22 (9.3)		
2-5	66 (28.0)		
6-10	85 (36,0)		
11-15	27 (11.4)		
≥ 16	32 (13.6)		
Number of different professions represented^*β*^			3 (1.3)
1	31 (13.1)		
2-5	127 (53.8)		
≥ 6	75 (23.0)		
*Number of organizations with following providers (n = 214)* ^*β*^ *:*		4.4 (2.6)	
Acupuncturist	50 (23.4)		3 (1.4)
Chiropractor	5 (2.3)		3 (1.4)
Family physician	17 (7.9)		3 (1.4)
Kinesiologist	62 (29.0)		3 (1.4)
Massage therapist	142 (66.4)		3 (1.4)
Occupational therapist	67 (31.3)		4 (1.9)
Orthopedist	32 (15.0)		3 (1.4)
Orthotist	36 (16.8)		3 (1.4)
Osteopath	106 (49.5)		3 (1.4)
Physiotherapy assistant	145 (67.8)		3 (1.4)
Psychologist	33 (15.4)		3 (1.4)
Sport physician	32 (15.0)		3 (1.4)
In physical proximity^ϕ^ with providers outside organization*	151 (64.0)		1 (0.4)
With vacant provider positions*	65 (27.5)		1 (0.4)
Presence of secretary (ies)/receptionist (s)^β^	207 (87.7)		1 (0.4)
PT access to*:			
Computer	219 (92.8)		
Internet	209 (88.6)		1 (0.4)
Electronic physiotherapy patient records	26 (11.0)		3 (1.3)
*Structure*			
Time since opening (years)^β^			
< 5	39 (16.5)		
5-10	36 (15.3)		
> 10	161 (68.2)		
Part of organizational network^β^	109 (46.2)		
Number of organizations in network (n = 109)			1 (0.9)
2-5	69 (63.3)		
6-10	15 (13.8)		
> 10	24 (22.0)		
Shared patient records^β^ (n = 212)	116 (54.7)		
Had available forms for interactions between providers^β^	168 (71.2)		1 (0.4)
Had rules regarding interactions between providers/organizations^β^	157 (66.5)		2 (0.8)
Types of rules regarding interactions (n = 157)			
Implicit	120 (76.4)		
Written	12 (7.6)		
Implicit + written	25 (15.9)		
Frequency of planned meetings to discuss clinical cases in previous 12 months^β^ (n = 213)			1 (0.5)
Daily	4 (1.9)		
Weekly	36 (16.8)		
Monthly	25 (11.7)		
A few times	70 (32.7)		
Never	78 (36.4)		
*Practices*			
Mean duration of physiotherapy assessments^β^ (minutes)		59.1 (8.1)	2 (0.8)
< 60	23 (9.7)		
60	199 (84.3)		
>60	12 (5.1)		
Mean duration of physiotherapy treatment sessions^β^ (minutes)		41.9 (12.8)	2 (0.8)
≤ 30	105 (44.5)		
> 30 < 60	69 (29.2)		
≥ 60	60 (25.4)		
Offered physiotherapy training^β^	105 (44.5)		1 (0.4)
*Types of services offered:*			
Electrotherapy	215 (92.7)		4 (1.7)
Hydrotherapy	104 (45.6)		8 (3.4)
Pediatric treatment for plagiocephaly/torticollis	103 (45.6)		(4.2)
Osteopathic approaches	139 (61.2)		9 (3.8)
Postural approaches	112 (50.2)		13 (5.5)
Neurological approaches	62 (27.8)		13 (5.5)
Sports physiotherapy	197 (84.9)		4 (1.7)
Work rehabilitation	119 (52.4)		9 (3.8)
Vestibular re-education	100 (45.2)		15 (6.4)
Manual therapy-mobilizations	224 (97.0)		5 (2.1)
Manual therapy-manipulations	147 (65.9)		13 (5.5)
Perineal re-education	67 (29.8)		11 (4.7)
Use of needles under the dermis	30 (13.6)		15 (6.4)
Exercise/education classes	69 (31.4)		16 (6.8)
Other	42 (17.8)		
Number of services offered*^ϵ^		7.3 (2.4)	15 (6.4)

*Identifies variables that were first included in the initial multiple correspondence analyses, but were not retained in these analyses.

^α^Scale 0–10 for each statement: “no importance” to “highest importance possible”.

^λ^PT = physiotherapist.

^χ^Inside or outside of organization.

^ψ^≥ 10 000 inhabitants [43].

^β^Identifies variables that were included in the initial multiple correspondence analyses, and were retained in these analyses.

^ϕ^Within 5 minutes walking distance.

^ϵ^Excluding “other” category.

Regarding structural aspects, the majority of organizations had been open for more than 10 years (68.2%) and were not part of a network or group of organizations belonging to the same owners (53.8%). In most organizations, patient records were shared by multiple providers (54.7%), forms were available for interactions with other providers (71.2%; e.g. letter template), and there were implicit or explicit rules regarding interactions with other providers or organizations (66.5%), such as when to send a letter to a physician or service agreements. Furthermore, planned meetings between providers to discuss clinical cases had never occurred or only a few times over the previous year in most organizations (69.1%).

Finally, in terms of practices, the majority of organizations offered multiple types of services (mean 7.3; SD = 2.4). The mean duration of physiotherapy evaluation and treatment sessions were respectively 59.1 (SD = 8.1) and 41.9 (SD = 12.8) minutes, and more than half of the organizations did not offer in-house physiotherapy training for future physiotherapists and physiotherapy assistants (55.1%).

### Organizational models

**Figure 2:**
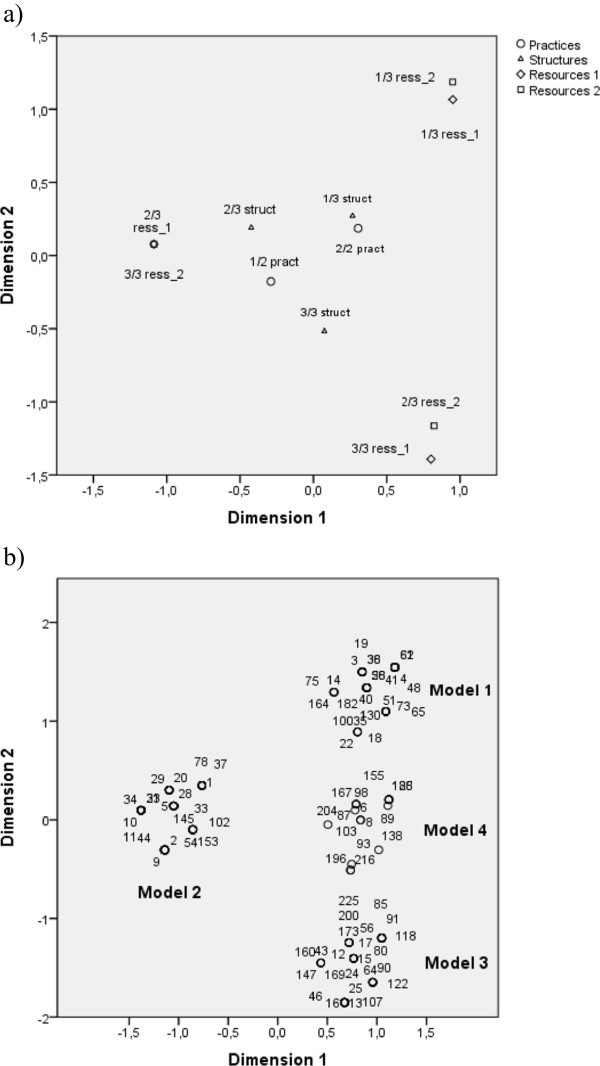
**Graphical representations of taxonomical analysis. a)** Localization of categories of dimensions; **b)** Corresponding localization of organizations and models.

The first multiple correspondence analyses conducted separately for the four organizational components led to the identification of four relevant dimensions: two for resources, one for structures, and one for practices (none for vision) (Figure 1). Using these four dimensions as the basis for the final multiple correspondence analyses allowed to identify four different organizational models, three of them being characterized by different organizational resources, labelled as solo practice, middle-scale multiprovider and large-scale multiprovider organizations (Models 1–3; Figure 2); the fourth one with all categories of practices and structure dimensions in rather close proximity (Model 4; Figure 2). The dominant characteristics associated with each model are presented in Table 3. Almost half of the organizations were best represented by Model 2 *middle-scale multiprovider* (44.8%), while Model 4 *mixed* represented only 9% of them (Table 3). The total percentage of explained variance obtained with the final multiple correspondence analyses was 52.3% for the first dimension and 43.8% for the second, for a total of 96.1%.

**Table 3:** **Organizational models (n = 201)**

Organizational model	Main characteristics of organizations	% of organizations
Model 1*-Solo practice*	o 1 provider	23.4
	o 1–2 types of providers*	
	o No secretary/ receptionist	
	o Located in provider’s home	
Model 2- *Middle-scale multiprovider practice*	o 2–10 providers	44.8
	o 3–6 types of providers	
	o Presence of secretary (ies)/receptionist (s)	
	o Located in building with organization only or multiple businesses/organizations	
Model 3- *Large scale multiprovider practice*	o ≥ 11 providers	22.9
	o ≥ 7 types of providers	
	o Presence of secretary (ies)/receptionist (s)	
	o Located in sport center/school or other location	
Model 4- *Mixed*	Practices/structure	9.0

*One provider could work under more than one professional title (e.g. physiotherapists and osteopath).

## Discussion

In this study, our objectives were to describe the characteristics of organizations where physiotherapists practice in the private sector, as well as to explore the existence of a taxonomy of organizational models, using a configurational approach encompassing organizational vision, resources, structure and practices.

The findings of this study provide a detailed picture of the organizations within which physiotherapists practice in the private sector. As mentioned previously, very little research has been carried out in this area until now, existing studies having focused on the professional’s themselves, not their workplaces. In a study on Québec physiotherapists’ professional practices conducted by Mikhail et al. [27], like in our study, the organizations where the 47 participating physiotherapists practiced in the private sector were mainly located in an urban area (91.5% in both studies). Many workplaces offered physiotherapy training for future physiotherapists (42.6% in Mikhail et al.’s study vs. 44.5% in our study that also included training for physiotherapy assistants), a proportion that can be viewed as rather low, considering that a large number of physiotherapy students ultimately work in the private sector. Most organizations had internet access with 74.5% in Mikhail et al.’s study vs. 88.6% in our study, possibly indicating increased access in the last few years) [27].

Our findings also highlight that most physiotherapists work in organizations with multipleproviders, including providers representing diverse professions. These organizations also offer a number of tools that are used for interactions with other providers, such as shared patient records, rules of functioning and standardised forms. This is in line with the promotion of interprofessional practices in health systems everywhere in recent years [44], including the rehabilitation field, notably for patient populations who frequently consult physiotherapists in the private sector, such as for low back pain [45, 46]. The trend towards increasing numbers of multidisciplinary rehabilitation clinics has been documented previously elsewhere in Canada [47]. The Federation of Private Practice Physiotherapists of Québec, that represents a subset of private physiotherapy clinics in the Province, has also supported the development and formalization of interdisciplinary work in recent years [48]. However, our results show that planned meetings were reported as mostly occurring infrequently. This strategy may require high formalization of interactions between providers and may be viewed as difficult to operationalize in a context where providers have varying schedules and high caseloads. This finding may also reflect the belief that planned meetings and access to multiple providers are easier in the public sector than in the private sector, observations that were mentioned by participants in our previous qualitative study.

In addition, this study allowed to identify four organizational models where private sector physiotherapists practice. Based on the results of our analyses, the most important variables defining organizational models were resource-related, namely number of providers and types of providers represented, presence of secretaries and specific work environment. Almost half of the organizations were best represented by an organizational model characterized by an intermediate number of providers and types of providers, the presence of secretaries and other organizations or businesses in the immediate environment. Most other organizations were either associated with an organizational model that could be referred to as home solo practice, or larger scale organizations. In a previous case study research, private for-profit clinics in Ontario were categorized in a different manner as sole owner independent, network-based independent, rehabilitation corporations and other types [12].

The importance of human resources in the organization of care was highlighted in previous research, although very rarely in the rehabilitation field, to our knowledge. The number and types of providers involved were also defining characteristics in Lamarche et al.’s [14] results on models of primary care organizations. The presence of secretaries as a central characteristic of the organizational models may reflect the fact that with higher numbers of providers in an organization, there inevitably is more administrative work to be accomplished, hence justifying the need for clerical resources. Interestingly, we observed that middle-scale organizations did not align vertically with the other models in Figure 2. This may be an indication that solo practice and large-scale organizations share a common feature, one that distinguishes them from middle-scale organizations, hence indicating that increasing an organization’s resources is not a pure linear process. Further research is however needed to help explain this aspect, as well as any defining features of the fourth organizational model identified. It is probable that this model is mostly associated with structure or practice-related features. Furthermore, even if organizations were identified as being best represented by one model, it does not imply that they all share identical characteristics [20]. This may be because of the dynamic nature of organizations and models, which may be in the process of transitioning from one model to another [20].

Having identified these organizational models has potential implications for further studying physiotherapy services and practices. Our results could be used to examine the influence of organizational models on physiotherapists’ professional practices and patient outcomes. As an example, we included these organizational models in another study looking at the factors associated with private sector physiotherapists’ interprofessional practices and found that the organizational model to which physiotherapists belonged was associated with the intensity of their interprofessional practices (submitted). Furthermore, having highlighted that resource-related variables are most important in distinguishing organizational models, one could potentially use these variables to develop an instrument to identify group membership of organizations. In another vein, this taxonomy could also be useful for stakeholders such as professional boards, associations, managers and decision-makers, in planning human resources deployment and development of physiotherapy services.

### Study limits

One of the main limits of this study is that the identification of organizational characteristics and models relied on data obtained through self-report, which may have produced information bias. Gathering other forms of data, such as those obtained by field observations or scanning organizational documents, could provide complementary data. The fact that the physiotherapists acted as respondents for organizational-level data, rather than the owners of the organizations, may have led to incorrect responses, although a good proportion of participating physiotherapists (34.3%) were also the owners of the organizations. Furthermore, based on pre-testing of our questionnaire, no difficulty was reported regarding completion of questions relating to organizational data by physiotherapists, and the proportion of missing values was very small.

As for data analyses, even though we attempted to be exhaustive, it is most probable that, in preparing this study, we omitted important variables that may play an important role in organizations and in defining organizational models. The choice of variables to include in our multiple correspondence analyses was influenced by the aim of our other related study of physiotherapists’ interprofessional practices and probably influenced the results. It is also possible that our categorizations of the variables retained in the multiple correspondence analyses did not perfectly match the nature of the data. Indeed, relationships between variables may not have emerged because our cut-off points between categories may have been inadequate. Other forms of analyses could have also been used to attempt to identify organizational models instead of multiple correspondence analyses. As an example, previous studies of organizational models in the healthcare field have been conducted using cluster analyses [20]. However, this type of analyses has also been associated with certain weaknesses [49]. Because of the dynamic nature of healthcare [19, 20], further studies should be conducted in order to validate our findings obtained through a cross-sectional study, as well as to capture changes in the organization of physiotherapy services. Moreover, while this study was conducted in one Canadian province (Québec), we believe they can possibly be generalized to similar contexts in other provinces and western countries where many physiotherapists also work in the private sector.

## Conclusions

To our knowledge, this was the first study to have drawn a detailed portrait of the organizations where physiotherapists practice in the private sector, as well as to have attempted to identify a taxonomy of organizational models. According to our results, most physiotherapists work in organizations regrouping multiple providers from diverse professions. Furthermore, four different organizational models were identified, with organizational resources being the main distinguishing features.

The results of this study offer new knowledge of interest for practicing physiotherapists and owners of the organizations where they practice, physiotherapy and other professional boards and associations, the health services and rehabilitation research communities, as well as policy-makers and deciders involved in planning physiotherapy and rehabilitation services. Further research examining the organization of physiotherapy in the private sector is nonetheless needed.
